# Supplementary material for: A new external jugular venipuncture technique for efficient vascular access that exploits a murine anatomical variation
Source: PLoS One. 2025 Sep 25;20(9):e0329811. doi: 10.1371/journal.pone.0329811 (PMC12463251; doi:10.1371/journal.pone.0329811)
Supplement: S1 File — The step-by-step protocol is also available on protocol.io. https://dx.doi.org/10.17504/protocols.io.eq2lywqkevx9/v1. (DOCX) [file pone.0329811.s001.docx]

**S1 File. Sternoclavicular joint-Targeted External jugular venipuncture Method (STEM).** The step-by-step protocol is also available on protocol.io. https://dx.doi.org/10.17504/protocols.io.eq2lywqkevx9/v1
