# Supplementary material for: A new external jugular venipuncture technique for efficient vascular access that exploits a murine anatomical variation
Source: PLoS One. 2025 Sep 25;20(9):e0329811. doi: 10.1371/journal.pone.0329811 (PMC12463251; doi:10.1371/journal.pone.0329811)
Supplement: S2 Text — The comprehensive imaging methodology implemented in this study and the experiments performed to establish the technique are described in detail. (DOCX) [file pone.0329811.s002.docx]

**S2 Text. Supplementary Methods**

**High-Resolution Ultrasound Evaluation**

C57BL/6J mice (n=12) were weighed prior to ultrasound examinations. Anesthesia was induced with isoflurane 3% and maintained at 1.5-2% during the examination. Prior to ultrasound imaging, the fur on the chest and neck of the mice was removed using a commercial depilatory cream (Nair, Church & Dwight Co., Inc., Ewing, NJ, USA) applied for 60 seconds and gently wiped off with warm, damp gauze. High-resolution ultrasound imaging was performed using a Vevo F2 system (FUJIFILM VisualSonics, Toronto, ON, Canada) equipped with a 50 MHz linear array transducer (MX700).

**Contrast CT Evaluation and image editing**

C57BL/6J mice (n=5) were weighed prior to CT scanning. Anesthesia was induced with 3% isoflurane and maintained at 1.5-2% during the evaluation. ExiTron™ nano 12000 (Viscover™, NanoPET Pharma GmbH, Berlin, Germany), an alkaline earth metal-based nanoparticulate contrast agent, was used for this study. All mice received 100 μL of Viscover™ via retro-orbital injection, equivalent to 1200 mg iodine/kg body weight. CT scans were performed using a NanoPET/CT small animal scanner (Mediso Medical Imaging Systems, Budapest, Hungary). Multi-planar reconstruction (MPR) images and 3D renderings were generated using Vivoquant^TM^ 2022 software (Invicro, Boston, MA, USA).

**Autopsy**

Twelve-week-old C57BL/6J mice (n=5), 8-week-old BALB/cJ, NOD/ShiLtJ and DBA2J (each n=2) underwent necropsy. The mice were euthanized by CO_2_ inhalation, the cervicothoracic region dissected, and the mice positioned in the supine position with both forepaws abducted (Fig 5A). The skin of the thoracic neck was removed, and the submandibular gland excised (Fig 5B). The left pectoralis major muscle was surgically excised to reveal a full view of the central portion of the external jugular vein. (Fig 5C, 5D, 6D, 6E, 6F). Subsequently, both clavicles, ribs, and sternum were removed to expose the thoracic cavity (Fig 5E). This approach allows visualization of the anatomical relationships between the major intrathoracic organs and the external jugular vein.

**Comparison of STEM and other venipuncture**

**Tail venipuncture (TV)**

C57BL/6J mice (n=10) were weighed prior to blood collection. The mice were placed in a restraint device to expose their tails. The operator cleaned the tail with an alcohol wipe, locating the lateral tail vein about one-third along the tail length from the tip. Carefully immobilizing the tail and rotating it slightly, the researcher uses a lancet or needle to puncture the vessel. Blood is collected using a capillary tube, with the amount varying based on the study requirements. After collection, gentle pressure is applied with gauze to stop the bleeding.

**Retro-Orbital Bleeding (ROB)**

C57BL/6J mice (n=10) were weighed prior to blood collection. Mice were anesthetized using isoflurane inhalation anesthesia. The operator protruded the eye by placing a finger on the top of the head and along the jawline, pulling the skin back and down, taking care not to apply pressure to the trachea. A microhematocrit tube is inserted at the medial canthus of the eye, directed caudally at a 30-45° angle from the plane of the nose, and gently rotated to cut through the conjunctival membranes and rupture the ocular plexus. The tubes were gently rotated to prevent blood coagulation and increase the efficiency of blood collection. To stop the bleeding, the skin is released, allowing the eye to return to its normal position, and pressure is applied to the orbit to ensure hemostasis.

**Corticosterone Measurement**

Plasma corticosterone levels in mice were measured using a Corticosterone Competitive ELISA Kit (Invitrogen, EIACORT) following the manufacturer's protocol. Blood samples were collected from 6 C57BL/6J mice each in TV, ROB, and STEM. Plasma was separated by centrifugation. Samples were diluted as necessary and added to the pre-coated microplate along with standards, conjugate, and antibody. After the incubation and washing steps, substrate solution was added, and absorbance was read at 450 nm. Corticosterone concentrations were calculated using a standard curve. All samples were analyzed in duplicate.

**Intravenous injection pharmacokinetics**

C57BL/6J mice (n=10) were weighed prior to the experiment. Mice were divided into two groups according to the route of IV administration: external jugular vein and lateral tail vein. Indocyanine green (MP Biomedicals, OH, USA) was dissolved in 1ml of purified water, of which 0.5mg/kg (ICG) was added to PBS to a total dose of 50µl and administered through each route of administration. The biodistribution of ICG in mice at different time points (1 min, 30 min, 60 min, 180 min and 360 min after administration) was determined using an IVIS Spectrum imaging system (Xenogen; Perkin Elmer, MA, USA). The system was set to an excitation wavelength of 745nm and an emission wavelength of 840nm. Images were analyzed using Living Image software. Configuration of parameters for imaging was the same for all mice.
