# Supplementary material for: A new external jugular venipuncture technique for efficient vascular access that exploits a murine anatomical variation
Source: PLoS One. 2025 Sep 25;20(9):e0329811. doi: 10.1371/journal.pone.0329811 (PMC12463251; doi:10.1371/journal.pone.0329811)
Supplement: S3 Table — Technical outcomes of STEM in BALB/cJ, NOD/ShiLtJ, and DBA/2J mice. (DOCX) [file pone.0329811.s003.docx]

**S3 Table. Validation of STEM to mouse strains other than C57BL/6J**

|  | BALB/cJ | NOD/ShiLtJ | DBA/2J | P value |
| --- | --- | --- | --- | --- |
| Sex (M:F) | 2:2 | 2:2 | 2:2 | N.S. |
| Body weight (g)* | 22.2±3.0 | 22.1±2.1 | 19.2±0.9 | 0.212 |
| # of Punctures** | 1 [1-1.75] | 1 [1-1.75] | 1 [1-1] | 0.577 |
| Procedure time (sec)* | 54±6.3 | 53±8.9 | 53±3.9 | 0.995 |
| Success rate of blood collection with a blood volume target of 0.5% body weight (%) | 100 | 100 | 100 | N.S. |
| Complication Rate (%) | 0 | 0 | 0 | N.S. |

* Values are given as the mean±standard deviation. ** Values are given as median [IQR]., One-way ANOVA was applied for body weight and procedure time, while the Kruskal-Wallis test was used for body weight and puncture time. Statistical significance was set at p < 0.05. N.S.: Not significant
