# Supplementary material for: A new external jugular venipuncture technique for efficient vascular access that exploits a murine anatomical variation
Source: PLoS One. 2025 Sep 25;20(9):e0329811. doi: 10.1371/journal.pone.0329811 (PMC12463251; doi:10.1371/journal.pone.0329811)
Supplement: Attachment — Submitted filename: Response to Reviewers.docx [file pone.0329811.s004.docx]

**Response to Reviewers**

March 3, 2025

Dear Emily Chenette, Editor-in-Chief, and Haipeng Liu, Academic Editor,

Thank you for inviting us to submit a revised draft of our manuscript entitled, “**A new external jugular venipuncture technique for efficient vascular access that exploits a murine anatomical variation**” to PLOS ONE. We also appreciate the time and effort you and each of the reviewers have dedicated to providing insightful feedback on ways to strengthen our paper. Thus, it is with great pleasure that we resubmit our article for further consideration. We have incorporated changes that reflect the detailed suggestions you have provided. We also hope that our edits and the responses we provide below satisfactorily address all the issues and concerns you and the reviewers have noted.

To facilitate your review of our revisions, the following is a point-by-point response to the questions and comments. Revisions in the manuscript are indicated in red text.

Reviewer #1: Comment 1

The study only used C57BL/6J mice. Expanding validation to other strains would enhance generalizability. Although the authors acknowledge this limitation, including preliminary data from additional strains could provide greater insights.

**Response: Thank you for providing these insights. We completely agree that this was a limitation, and so we now have extended our experiments to three additional strains of mice: BALB/cJ, NOD/ShiLtJ, and DBA2J. As a result, we are now able to demonstrate clearly the anatomical similarities of the external jugular vein among different mouse strains and the generalizability of STEM as a versatile technique. Please see the relevant section of the revised manuscript (Lines 216-218, 271-277, 336-337, 474-481; Fig 6 and S2 Table)**

Reviewer #1: Comment 2

The manuscript reports means and standard deviations, but the learning curve data would benefit from inferential statistical tests (e.g., ANOVA) to determine the significance of differences between phases.

**Response: Thank you for your suggestion. We have now performed the appropriate statistical analysis in the relevant sections (Lines 178-189, 329-334 and Table 1).**

Reviewer #1: Comment 3

Additionally, sections comparing EJV and tail vein injections could use more rigorous statistical analysis to confirm the lack of significant differences.

**Response: Thank you for your suggestion. We have incorporated your comments and have defined and analyzed the region of interest (ROI) by IVIS in order to clarify the differences in pharmacokinetics between the two IV administration methods. Please see the relevant changes in the revised manuscript (Lines 433-438, 445-453, 458-460 and 466-472.**

Reviewer #1: Comment 4

While the advantages over lateral tail vein injection are discussed, the manuscript could include direct comparisons of stress markers or physiological outcomes between methods. Evaluating the performance of STEM against other venous access points (e.g., retro-orbital bleeding) would further underscore its strengths.

**Response: Thank you for providing these valuable insights. We have incorporated your comments and made a direct comparison between surgical outcomes and plasma corticosterone measurements as a stress biomarker for STEM, Tail venipuncture (TV) and Retro-orbital bleeding (ROB). While we cannot completely rule out variance in the data due to the skill and experience of the operator, your suggestion of additional data does help to strengthen our argument. Please see the relevant edits in the revised manuscript (Lines 372-392, 394-400, 402-406, Fig 7 and Table 3)**

Reviewer #2: Comment 1

The introduction effectively outlines the challenges of venous access in mice but does not adequately justify why the STEM method is superior to current techniques such as lateral tail vein or retro-orbital venipuncture. A more thorough literature review on existing venous access methodologies would provide a stronger rationale for this study.

**Response: Thank you for your thoughtful review. We have added a literature review of current blood collection methods where applicable (Lines 90-117).**

Reviewer #2: Comment 2

The study lacks detail on how anatomical variations across different mouse strains might impact the reproducibility of the technique. Validation in non-C57BL/6J strains is critical for generalization.

**Response: Thank you for providing these insights. We completely agree and performed additional experiments on the mouse strains - BALB/cJ, NOD/ShiLtJ, and DBA2J. As a result, we were able to confirm the anatomical uniformity of the external jugular vein and the reproducibility of our STEM procedure among different mouse strains. As you pointed out, these new data provide strong evidence for the generalizability of our technique. Please see the relevant sections of the revised manuscript (Lines 216-218, 271-277, 336-337, 474-481; Fig 6 and S2 Table)**

Reviewer #2: Comment 3

Provide more information on the sample size calculation and statistical power analysis to ensure that the study is sufficiently powered to draw conclusions. The manuscript should explicitly state whether operators were blinded during the evaluation of success rates, procedural time, or complication rates.

**Response: Thank you for pointing out such an important deficiency in the manuscript. We have added a description in the relevant section (Lines 313-124, 316-322, and 349-355).**

Reviewer #2: Comment 4

The results emphasize the efficiency of STEM but do not compare this method against other established techniques in a head-to-head analysis. Including data on procedural success, time, and complications relative to existing methods would provide stronger evidence of STEM's advantages.

**Response: Thank you for providing these valuable insights. We performed a head-to-head analysis of STEM and other established methods, tail venipuncture (TV) and retro-orbital bleeding (ROB), with respect to procedural outcomes. Thanks to this suggestion, we now have evidence for the procedural superiority of STEM. Please see the relevant edits in the revised manuscript (Lines 372-392, 394-400, Fig 7 and Table 3)**

Reviewer #2: Comment 5

The training outcomes for novice users are underwhelming. The mean procedure time of 281 seconds with 2.8 attempts indicates that further optimization or training protocols may be required to ensure broader adoption.

**Response: Thank you for pointing this out. Your perspective is very important as we ponder how to show the general appeal of STEM to other researchers. We realize that we may not have addressed all the procedural learning and mastery issues at this time. We have added to the Discussion, comments describing the current limitations of training novices in the study and as a subject for future research (Lines 485-490).**

Reviewer #2: Comment 6

The lack of significant differences in pharmacokinetics between the external jugular and lateral tail vein administrations raises questions about the added value of STEM beyond ease of use.

**Response: Thanks for pointing that out. We don’t consider STEM's lack of pharmacokinetic differences in IV administration from the tail vein necessarily to be negative. Rather, the fact that STEM has demonstrated pharmacodynamics similar to IV administration from other established methods such as tail vein injection suggests that STEM can be applied to IV administration as well as blood collection. Moreover, STEM may be viewed as an alternative route of administration to a tail vein especially given its numerous benefits.** **We have added a description of this perspective, which we did not fully explain, in the relevant section (Lines 445-453 and Fig 8).**

Reviewer #2: Comment 7

The discussion should include a more balanced assessment of the limitations of STEM, particularly its applicability to other mouse strains and potential operator variability.

**Response: We agree with your point. We have been able to add results using other mouse strains and have updated the discussion accordingly. We have also included a reference to the variability in technique depending on the surgeon (Lines 474-481 and 485-490).**

Reviewer #2: Comment 8

Address the ethical implications of central venous access in mice, particularly concerning potential pain and stress despite claims of minimal sedation requirements.

**Response: Thank you for your suggestion. A comparative analysis of plasma corticosterone measurements as a stress marker among multiple blood collection methods, including STEM, is now included and demonstrates the validity of central venous access. We have added that description in the relevant section (Lines 387-392, Fig 7).**

Reviewer #2's Comment 9

While the study emphasizes the potential for STEM to replace traditional methods, there is insufficient evidence to support its widespread adoption without further comparative and validation studies.

**Response: Thank you for providing these valuable insights. We performed an experiment comparing STEM as a traditional blood collection method with tail venipuncture and retro-orbital bleeding and added the results of surgical outcomes and stress levels addressed in Comment 9. Although these results do not completely eliminate bias due to the surgeon's skill level and experience, we believe that they may provide some evidence for the feasibility of STEM on a broader scale. Please see the relevant section of the revised manuscript (Lines 372-392, 485-490, Fig 7 and Table 3)**

Reviewer #2's Comment 10

The conclusion should temper claims of STEM’s superiority and emphasize the need for further validation studies to establish its generalizability and translational relevance.

**Response: Thank you for your insightful comments. We were able to revise the conclusion of the manuscript with more appropriate phrasing (Lines 491-494).**

We hope the revised version is now suitable for publication and look forward to hearing from you in due course.

Sincerely,

Malcolm V. Brock, M.D.

The E.F. Gordon Professor of Surgery and Oncology
