# Supplementary material for: A new external jugular venipuncture technique for efficient vascular access that exploits a murine anatomical variation
Source: PLoS One. 2025 Sep 25;20(9):e0329811. doi: 10.1371/journal.pone.0329811 (PMC12463251; doi:10.1371/journal.pone.0329811)
Supplement: Attachment — Submitted filename: R2 Response to Reviewers.docx [file pone.0329811.s005.docx]

Response to Reviewers

August 22, 2025

Dear Editors,

Thank you for inviting us to submit a revised draft of our manuscript entitled, “**A new external jugular venipuncture technique for efficient vascular access that exploits a murine anatomical variation**” to PLOS ONE. We also appreciate the time and effort you and each of the reviewers have dedicated to providing insightful feedback on ways to strengthen our paper. Thus, it is with great pleasure that we resubmit our article for further consideration. We have incorporated changes that reflect the detailed suggestions you have provided. We also hope that our edits and the responses we provide below satisfactorily address all the issues and concerns you and the reviewers have noted.

*Reviewer #3: The manuscript presents a protocol with clear utility to the research community, addressing a common procedural challenge in mouse studies and filling a gap in the published literature. The method is described in sufficient detail for an experienced researcher to reproduce it, with clear step-by-step guidance, figures, and methodological descriptions. Validation is supported by original data on procedural success rates, pharmacokinetic equivalence to tail vein injection, and stress assessment via corticosterone, though the scope is limited to healthy adult mice in one laboratory. All underlying data are made fully available in the supporting information, consistent with the PLOS Data policy. The manuscript is written in clear, standard English and is easy to follow, with only minor opportunities for improving readability by breaking up long sentences in the Discussion and briefly clarifying less common anatomical terms.*
 *Below are specific key points which can further enhance the manuscript’s credibility.*

*1. The paper mentions a small sample size but does not explain that some key analyses may not have had enough statistical power to detect real differences. This includes the pharmacokinetic test with five animals per group, the corticosterone test with six animals per method, and the procedural comparison with ten animals per method. While post hoc group comparisons were done, there was no power calculation to confirm these sample sizes could support claims of no difference. Without this clarification, readers might take non-significant results as proof the methods are equivalent. I understand these numbers may reflect practical constraints such as animal welfare regulations, resource limits, or the exploratory nature of the study. Ideally, the authors could add a short post hoc power estimate for these results. If that is not possible, they should at least state clearly in the discussion that these specific analyses were underpowered and that a lack of significance should be interpreted with caution.*

**Response:
We thank the reviewer for this constructive feedback. We fully agree with the reviewer’s concern. Due to ethical and logistical considerations in animal use, our group sizes were limited, and we acknowledge that several comparisons (e.g., pharmacokinetics, corticosterone, and success rate of procedural outcomes) may have been underpowered. We have revised the Discussion to explicitly state that these results should be interpreted cautiously and that non-significant differences do not conclusively demonstrate equivalence (Lines 469-475).**

*2. The study does not mention that all the experiments were carried out in a single laboratory. There is no discussion of whether the method would perform the same way in other facilities with different operators, equipment, or handling practices. Techniques that depend on identifying anatomical landmarks can be sensitive to user experience and handling style. Without inter-laboratory testing, it is unclear how reproducible the method would be elsewhere. I understand that expanding the work to multiple sites can be challenging due to coordination, time, and resource limits, especially in early-stage studies. Ideally, the authors could encourage future cross-lab testing to confirm reproducibility. If this cannot be added, at least noting in the discussion that this was a single-institution study would help set appropriate expectations for readers.*

**Response:
We thank the reviewer for raising this important point. As you pointed out, assuring the reproducibility of a lab-independent technique is important to demonstrate the general adaptability of STEM. We have revised the Discussion to emphasize this limitation and to note that inter-laboratory validation will be essential in the future (Lines 480-484).**

3. The study relies only on corticosterone levels to assess stress. While this is a standard and practical acute stress biomarker, it does not capture behavioral, recovery, or long-term welfare aspects. A single physiological measure can miss other impacts on the animals, and a more complete welfare profile would strengthen the method’s ethical claims. I understand that adding behavioral or long-term measures would require extra time, staff, and possibly new ethical approvals, which may not have been feasible in this study. Ideally, the authors could note this limitation and suggest that future work includes additional welfare indicators. At the very least, the discussion could explain why corticosterone was chosen as the sole measure and state that it provides only a partial picture of welfare.

**Response:**

**We appreciate this suggestion. As noted by the reviewer, corticosterone was selected as a practical biomarker of acute stress, but it does not capture behavioral or long-term effects. We have clarified in the Discussion that corticosterone alone gives only a partial picture and suggested that future studies integrate behavioral and long-term indicators of animal welfare (Line 368, Lines 373-376).**

4. The method was tested only in healthy adult mice from four strains. There is no discussion of whether it would work as well in juvenile, aged, obese, or diseased animals, where anatomy or vein accessibility may differ. Applicability to a wider range of ages and health conditions is important for a method proposed for broad use. Although the study did not state this age group as an objective, using healthy adults is a common choice for proof-of-concept work because they are physiologically stable and widely used in research. Still, it would help to note clearly that these results may not apply to other life stages or disease models, and to suggest that future studies test the method under those conditions. This would better define the scope and limits of the current findings.

**Response:
We agree with this limitation and have added text to clarify the restricted scope of our study and the need for validation in other physiological contexts (Lines 466-469).**

*5. The pharmacokinetic results showed no significant difference between STEM and tail vein injection. In the discussion, this is framed as a positive finding, suggesting STEM is equally suitable for IV drug administration. However, it is not acknowledged that this also means STEM offers no pharmacokinetic advantage over the comparator. Interpreting a lack of difference only as a benefit could give an incomplete picture of the findings. Though showing equivalence is still valuable, it would help to note explicitly that STEM does not alter drug kinetics compared to tail vein injection, so its advantages are likely practical (ease, consistency, reduced handling stress) rather than pharmacological. This clarification would make the conclusions more balanced and transparent.*

**Response:
We appreciate this insightful point. We have revised the Discussion to clarify that STEM confers no pharmacokinetic advantage over tail vein injection, and that its benefits are primarily practical—such as ease of use, consistency, and potentially reduced handling stress (Lines 432-434, Lines 436-438). Thanks to you, the conclusion could be stated as a more transparent, balanced, and neutral interpretation.**

We hope the revised version is now suitable for publication and look forward to hearing from you in due course.

Sincerely,

Malcolm V. Brock, M.D.

The E.F. Gordon Professor of Surgery and Oncology
